# Enteric glia as a player of gut-brain interactions during Parkinson’s disease

**DOI:** 10.3389/fnins.2023.1281710

**Published:** 2023-11-01

**Authors:** Beatriz Thomasi, Luisa Valdetaro, Maria Carolina Ricciardi, Marianna Gonçalves de Carvalho, Isabela Fialho Tavares, Ana Lucia Tavares-Gomes

**Affiliations:** ^1^Department of Physiology, Michigan State University, East Lansing, MI, United States; ^2^Department of Molecular Pathobiology, NYU College of Dentistry, New York, NY, United States; ^3^Neuroglial Interaction Lab, Neuroscience Program, Universidade Federal Fluminense, Niterói, Brazil; ^4^Neuroglial Interaction Lab, Neurobiology Department, Universidade Federal Fluminense, Niterói, Brazil

**Keywords:** enteric glia, gut-brain axis, neuroglial signaling, neuroinflammation, Parkinson’s disease

## Abstract

The enteric glia has been shown as a potential component of neuroimmune interactions that signal in the gut-brain axis during Parkinson’s disease (PD). Enteric glia are a peripheral glial type found in the enteric nervous system (ENS) that, associated with enteric neurons, command various gastrointestinal (GI) functions. They are a unique cell type, with distinct phenotypes and distribution in the gut layers, which establish relevant neuroimmune modulation and regulate neuronal function. Comprehension of enteric glial roles during prodromal and symptomatic phases of PD should be a priority in neurogastroenterology research, as the reactive enteric glial profile, gastrointestinal dysfunction, and colonic inflammation have been verified during the prodromal phase of PD—a moment that may be interesting for interventions. In this review, we explore the mechanisms that should govern enteric glial signaling through the gut-brain axis to understand pathological events and verify the possible windows and pathways for therapeutic intervention. Enteric glia directly modulate several functional aspects of the intestine, such as motility, visceral sensory signaling, and immune polarization, key GI processes found deregulated in patients with PD. The search for glial biomarkers, the investigation of temporal–spatial events involving glial reactivity/signaling, and the proposal of enteric glia-based therapies are clearly demanded for innovative and intestine-related management of PD.

## 1. Introduction

Parkinson’s disease (PD) is a neurodegenerative disorder that affects multiple systems. An avenue of scientific and clinical evidence brought rise to the interplay between the central nervous system (CNS) and the gastrointestinal (GI) tract, revealing what we know as the gut-brain axis. It is known that patients with PD present a range of GI pathological manifestations such as chronic constipation (affecting ~80% of patients with gastrointestinal symptoms), abdominal pain, and other signs of disorders of gut-brain interaction (DGBI) that can be present before any classic motor symptom (Travagli et al., 2020; Moudgal et al., 2021). Several groups have postulated theories for the pathogenesis of PD and, in an attempt to define a beginning, the truth is that today we have evidence to understand PD as a highly heterogeneous pathology that must involve different neuroinflammatory and neurodegenerative pathways (Leclair-Visonneau et al., 2020; Rolli-Derkinderen et al., 2020). Even subtypes of PD (body-first or brain-first) based on clinical and post-mortem evaluation have been proposed (Borghammer et al., 2022). In both ways, the gut-brain axis is recurrently described as an integral part of the PD, and a pathway that connects the different areas of the body where the disease manifests itself (Chen and Lin, 2022; Tan et al., 2022). This axis is subject to enormous modulation given the diversity of signaling at the level of the GI tract and the enteric nervous system (ENS; Carabotti et al., 2015). In the last years, a complex interaction between epithelial, neural, immunological, and microbial components emerged as critical factors in the pathology of PD.

In the GI tract, enteric glia play multiple functions and are recognized for their crucial role in GI physiology (D’Antongiovanni et al., 2023). These specialized cells of the ENS maintain intestinal homeostasis through crosstalk with various enteric cell types such as neuronal, epithelial, immune, and endocrine cells. The perception and modulation of the gut environment by enteric glia is based on a distinct transcriptional signature among glial types, which, associated with its morphological and locational diversity, makes enteric glia a unique glial type (Rosenberg and Rao, 2021). These cells are directly related to pathological conditions, such as DGBI, acting on the physiopathology of gut inflammation, dysmotility, and abdominal pain processes – known features of PD (Seguella et al., 2019; Lucarini et al., 2021; Rosenberg and Rao, 2021; Seguella and Gulbransen, 2021). In addition, evidence suggests that enteric glia are sensitive to gut microbiota and then contribute to gut-brain communication via microbial signaling pathways (Seguella et al., 2023). Treatments with commensal microbiota in animal models of PD have been shown to improve conditions associated with PD pathology, so the understanding of its interactions and mechanisms is fundamental.

This review aims to demonstrate the potential roles of enteric glia in the gut-brain axis in PD. Here, we give special attention to enteric glia phenotypes associated with glial neuroimmune modulation and inflammation, how glial and/or microbial molecules can affect each other, impacting gut dynamics, and the role of enteric glia in enteric neurotransmission, dysmotility, and visceral hypersensitivity. All these aspects, at different stages of the disease, likely contribute to the development and/or progression of PD and significantly impact patient quality of life. We gathered clinical data and animal models of PD, using different approaches, which allowed us to discuss and propose classic and new ideas to treat PD based on glial signaling.

## 2. Neuroinflammation through the gut-brain axis in Parkinson’s disease: the role of enteric glia

PD is a neuroinflammatory disorder in which inflammation has a key role in the neurodegenerative response (Tansey et al., 2022). Considering this, the interaction between gut neural and immune cells may contribute to generating the peripheral inflammatory response. Additionally, a complex enteric neuro-glial network participates in immune response modulation (Schneider et al., 2022). Studies have highlighted the gut condition in PD, in both patients and experimental models, suggesting it is a target for disease intervention and a source of pathologic/inflammatory signs related to PD pathogenesis (Travagli et al., 2020). Accordingly, it is well known that inflammatory bowel disease (IBDs) patients are at risk for PD (Lee et al., 2021; Li et al., 2023). Breakdown of the intestinal epithelial barrier (IEB) and gut inflammation has been described during PD, along with the changes in the enteric neurochemical code and enteric glial reactivity (Clairembault et al., 2014, 2015; Corbille et al., 2016; Coletto et al., 2021; Thomasi et al., 2022). Therefore, regardless of whether PD originates bottom-up or top-down through the gut-brain axis, peripheral neuroinflammatory responses are known to be associated with the disorder. There is ongoing debate regarding the role of peripheral neuroinflammation in promoting or exacerbating PD pathology through the gut-brain axis. Some reports show that intestinal inflammation induces and exacerbates neuroinflammation and dopaminergic neurodegeneration in CNS with or without detectable Lewy pathology (Garrido-Gil et al., 2018; Kishimoto et al., 2019; Lin et al., 2022). In this frame, the role of glial cells has been appreciated in the last decade and specifically enteric glia gained a starring role in the intestinal inflammatory scenario in PD as a key neuroimmune element.

Enteric glia is widely distributed throughout the GI tract, found in all layers, mucosal, submucosal/myenteric plexus, and muscular layers, and plays critical site-specific functions, essential for tissue physiology. Along with its original role of providing trophic supply to neurons, enteric glia is necessary for maintaining intestinal epithelium homeostasis, modulating intestinal motility, neurotransmission, and immune response (Gulbransen et al., 2012; McClain et al., 2014, 2015; Delvalle et al., 2018b; Grubisic et al., 2020; Ahmadzai et al., 2021; Seguella and Gulbransen, 2021). In PD, these functions are disrupted (Devos et al., 2013; Clairembault et al., 2014, 2015) and appear together with enteric glial reactivity, a hallmark of neuroinflammation. Evaluation of samples obtained by colonoscopy from patients with PD shows an association between the presence of reactive enteric gliosis, identified by the increase in glial markers glial fibrillar acidic protein (GFAP) and Sox10, together with the increase in proinflammatory cytokines such as IL-6, TNF-α, IFN-γ, IL-1β, and chemokines as CCL2 and CCL5 (Devos et al., 2013; Perez-Pardo et al., 2019). Such alterations contribute to impaired IEB integrity and increased permeability observed in individuals with PD (Forsyth et al., 2011; Perez-Pardo et al., 2019).

Animal models of PD have been very useful in demonstrating the participation of the gut-brain axis and postulating temporal–spatial characteristics and mechanisms. In other words, checking areas of the nervous system as well as layer-specific responses in the intestine and their possible interactions. Multiple groups have demonstrated that enteric glia is activated in the myenteric plexus in several types of animal models of PD, whether bottom-up or top-down models (Perez-Pardo et al., 2019; Dodiya et al., 2020; Pellegrini et al., 2020, 2022; Palanisamy et al., 2022; Thomasi et al., 2022). The bottom-up (or body-first) hypothesis postulates the body periphery, mainly the ENS, as the first site of pathologic processes that would be spread out until CNS via inflammatory and anatomical pathways as it progresses. In contrast, the top-down theory (or brain-first) says the genesis of PD would start in some areas of the CNS and then reach the body periphery (Borghammer and Berge, 2019; Borghammer et al., 2022). Although there are no detailed descriptions of the pathway that reverberates pathological signals from one pathogenic site to the other, some have been postulated to be happening through peripheral nerves, such as the vagus nerve (Anselmi et al., 2017; Nanni and Travagli, 2020). Our group recently demonstrated that enteric glial reactivity occurs quickly and early in a top-down mouse model of PD (Thomasi et al., 2022). Within the colonic neuromuscular compartment, only 48 h after injury by 6-hydroxydopamine (6-OHDA), we observed a rise in GFAP content. This was followed by histological markers of inflammation, an increase in mucosal TNF-α, and changes to the tight junction protein occludin (Thomasi et al., 2022). In addition, higher immunolabeling of GFAP in the mucosal layer was verified in the PD model 1 week after neurodegeneration induction (Thomasi et al., 2022). These findings highlight the early role of enteric glia in gut neuroinflammation induced by central neurodegeneration. This is in accordance with data in patients showing that colonic inflammation and glial pathologic recruitment are probably higher at the beginning of the disease course, so early events can be associated with GI (such as constipation) and CNS symptoms (like anosmia, sleep disturbances, mood disorders) development. In accordance, gut barrier sensitization, which is also an early pathologic event in PD (Pellegrini et al., 2022), is commonly verified in parallel to a higher permeability in the blood–brain barrier. This last one promotes microgliosis, damage to central neurons, and systemic inflammation set (Chen and Lin, 2022). Interestingly, GI and CNS symptoms such as constipation, depression, and sleep disturbances can appear years before advanced neurodegenerative processes in the CNS—which highlights the pre-motor phase. Low-grade inflammation is hypothesized as a current process that can underlie the prodromal phase of PD (Houser and Tansey, 2017; Rolli-Derkinderen et al., 2020).

Another relevant enteric glia feature is their immunocompetence since it can respond to damage through the activation of Toll-like receptors (TLR) like TLR2 and TLR4 (Turco et al., 2014). These same receptors are altered in PD, whether in the CNS or ENS and likely contribute to the early intestinal pathology identified in PD (Gorecki et al., 2021; Zhang et al., 2023). Moreover, enteric glia express MHC class II molecules, enabling communication with cells of the innate and adaptive immune systems (Da Silveira et al., 2011; Turco et al., 2014). Thus, enteric glia can detect signals present in the intestinal environment caused by the breakdown of the IEB. These signals can include microorganisms and microbial products, but also proinflammatory cytokines and neurotransmitters that bind to their receptors on enteric glia. In turn, enteric glia release proinflammatory cytokines, chemokines, and gliotransmitters like ATP, which contribute to the inflammatory response and neuronal damage (Chow and Gulbransen, 2017; Seguella and Gulbransen, 2021). Altogether, research about enteric glia during PD points to a clear relation with peripheral inflammation in PD, and this may favor the establishment of a general inflamed environment. Peripheric signaling likely reaches CNS through immune pathways and/or neuroglial signaling, and in both scenarios, enteric glia have quite a protagonism in the early phase of PD by their early reactive phenotype.

## 3. Enteric glia as a sensor of the gut microbiome

The gut-brain axis is considered an integral part of the PD pathophysiological processes, as demonstrated through several animal models and clinical correlations in patients (Blandini et al., 2009; Pellegrini et al., 2016a,b; Morais et al., 2018; Perez-Pardo et al., 2018; Dodiya et al., 2020; Nanni and Travagli, 2020; Thomasi et al., 2022; Xing et al., 2023). In addition, the intestinal microbiota is an important modulator of gut-brain signaling. It presents itself in dysbiosis, that is, an imbalance due to the reduction of beneficial commensal bacteria and alteration of their microbial metabolites such as short-chain fatty acids (SCFA) and lipopolysaccharide (LPS; Huang et al., 2023; Zhang et al., 2023). Due to the leaky gut condition, the ENS is exposed to pathological stimuli from the microbiota which may contribute to synucleinopathy, an alteration in α-synuclein metabolism that generates intracellular aggregates named Lewy bodies (Fitzgerald et al., 2019). Lewy bodies have been described both in the mucosal lamina propria and submucosal plexus (Wakabayashi et al., 1988; Corbille et al., 2016). However, which signaling pathways and cellular interactions are under this pathogenesis still needs to be better understood. In the last years, glia-microbe interactions have been addressed and defects in this signaling, as supposed to occur during PD, may drive ENS and CNS neuroimmune plasticity impacting neural function.

The mucosal enteric glia are in contact with multiple cell types such as immune-effector cells, enteroendocrine cells, blood vessels, and epithelium, thus performing intercellular communication in an integrative way that is critical for mucosal homeostasis (Bohorquez et al., 2014). This glial population is strongly influenced by microbiota, as mice treated with antibiotics and germ-free mice show mucosal glia depletion (Kabouridis et al., 2015). To understand the mechanism of this interaction, transgenic mice for fate-mapping show a new pool of enteric glia is recruited to the mucosal plexus from the myenteric ganglia, so the global enteric glia dynamic is sensitive to microbiota signaling (Kabouridis et al., 2015). This glial replenishment dynamic has been tested using human mucosal enteric glial cells xenografted in immune-compromised mice but it did not show dependence on the microbiome when tested through antibiotic treatment (Inlender et al., 2021). However, this has not been tested during inflammation or PD models, which provoke enteric glia reactivity and epithelial modifications in the gut barrier, as previously mentioned. The glial network of the mucosa closely regulates the intestinal epithelium, mainly in pathological conditions (Cheadle et al., 2014; Langness et al., 2017; Grubišić et al., 2022; Prochera and Rao, 2023), and *in vitro* studies demonstrate the glioprotective capacity on the intestinal epithelium via glia-derived s-nitrosoglutathione against acute exposure to bacterial pathogens (Flamant et al., 2011). S-nitrosoglutathione is a modulator of barrier function, mainly during inflammation, reinforcing tight junction function and integrity (Savidge et al., 2007; Flamant et al., 2011). Therefore, glia-driven effects due to microbial signaling may form a loop in the pro-inflammatory environment described in the mucosal layer of PD patients that accounts for intestinal epithelial plasticity.

The ability of the microbiota to drive glial plasticity adds complexity to Parkinsonian gut processes since profound modifications in the intestinal biofilm may be based on neuroimmune interactions established in the gut. Several microbial products, such as LPS and SCFA, could be candidates to promote signaling pathways associated with inflammation and cell metabolism, and those have been altered in patients with PD (Keshavarzian et al., 2015; He et al., 2020; Metzdorf and Tönges, 2021). When subjected to LPS treatment, a well-known damage-associated molecular pattern, enteric glia exert similar antigen-presenting cell features as MHC-II, CD86, glial derived neurotrophic factor (GDNF), TLR-2, and TNF-α expression (Yang H. et al., 2022). As mentioned, TLR signaling by activation of enteric glia can be a critical mechanism to integrate microbial signaling into ENS function (Gorecki et al., 2021). Interestingly, the glial protein S100B also comprises a signaling pathway that integrates glial TLR activity leading to NO release, an inhibitory neurotransmitter relevant to neuroglial interaction, motility, and inflammatory processes (Turco et al., 2014; Thomasi and Gulbransen, 2023). Seguella and colleagues argue that this glial signaling could be a defense response against bacterial bioproducts and pathogenic bacteria based on the acquisition of a glial pro-inflammatory profile that would collaborate for ENS dysfunction, inflammation, and dysbiotic reinforcement (Seguella et al., 2023). Both S100B and NO are molecules implicated in central and enteric neuroplasticity during PD (Colucci et al., 2012; Pellegrini et al., 2016a; Sampath et al., 2019; Angelopoulou et al., 2021; Kim et al., 2023). This TLR-S100B pathway has enormous modulatory potential for enteric function, mainly in the neuroimmune interactions established by enteric glia.

Bidirectionally, enteric glia is associated with microbial diversity based on structural domains of S100B (EF-hand calcium-binding and S100) and its protein domains. Interestingly, normal S100B condition and inhibition of its active or binding domain produce opposing effects on microbial diversity (Romano et al., 2021; Spica et al., 2023). Surprisingly, this work shows that distinct concentrations of S100B are associated with different microbial clusters. An *in silico* approach applying bioinformatics also indicated that the microbiota of healthy individuals and patients with IBDs might interact differentially with the multiple domains of S100B (Orsini et al., 2020). Then, they propose S100B as a candidate signaling molecule in the gut able to change microbial dynamics. Evidence from a unique cohort of PD patients shows that while mRNA of GFAP and Sox10 are upregulated in the colon, the levels of S100B are not modified (Devos et al., 2013). However, the gliotic profile that enteric glia assume during colonic inflammation in PD may also contribute to distinct protein–protein interactions and downstream signaling. S100B polymorphisms and their contributions to the pathogenesis of PD are widely described and tested at the CNS level (Angelopoulou et al., 2021) and, given the gut-brain axis, theories of genesis and propagation of the disease and glial signaling in the axis (Esposito et al., 2017; Borghammer and Berge, 2019; Leclair-Visonneau et al., 2020; Horsager et al., 2022), it is necessary to deepen the evaluation of enteric S100B.

Among the changes in the microbiome during PD, the variation of the *Bifidobacterium*, *Lactobacillus*, and *Akkermansia* genera stands out—being those recognized genera by the SCFA production (Romano et al., 2021). Studies with the reintroduction of SCFAs as butyrate or propionate in different animal models of PD demonstrate significant improvements in motor and cognitive deficits and IEB permeability (Kakoty et al., 2021; Liu et al., 2022). *Bifidobacterium* genus especially appears to impose an immune regulation on enteric glia that is variable among bacteria of this genus (Yang Y. et al., 2022). Both act on enteric glia to constrain or promote inflammation through receptor and neurotrophic factor modulation (Yang et al., 2020; Yang H. et al., 2022). Meanwhile, enteric glial responsiveness to SCFA may come from transporters and receptors activity and it seems that butyrate relies on glial internalization to activate the phosphokinase C (PKC) pathway (Defries and Beltran, 2020). Glial PKC in the gut is related to the capacitive feature of enteric glia that in turn evokes glial network and neuroglial communication (Sarosi et al., 1998). *Bifidobacterium* has also been correlated to improving the IEB by tight junctions modulation—a probiotic-based therapy proposed for PD patients (Tan et al., 2021; Di Vito et al., 2022). Interestingly, *Bifidobacterium animalis* can regulate GFAP and GDNF, glial proteins associated with the state of the IEB (Liu et al., 2022). In parallel, when treated with distinct *Bifidobacterium* species, the MPTP animal model of PD improved the CNS oxidative stress condition, protected the gut barrier, and suppressed the glial hyperactivation state (Li et al., 2022). This data demonstrates a clear connection between glial “immune” status with outcomes on microbiota and glial network that can ameliorate pro-inflammatory aspects of the gut-brain axis in PD.

## 4. The potential contribution of glial signaling in constipation and abdominal pain during Parkinson’s disease

Non-motor symptoms of PD include other dysfunctions that are credited to the primary condition of constipation, such as abdominal pain (Skjærbæk et al., 2021). Constipation is primarily a neuromuscular dysfunction involving impaired enteric neural signaling leading to slowing and/or incoordination of bowel movements (Metta et al., 2021). This dysfunctional pattern is described as a moderate sensitivity sign for future PD development (Mahlknecht et al., 2015). 28%–80% of PD patients experience constipation at some point in the pathological course (Klingelhoefer and Reichmann, 2017; Carrasco et al., 2018). Inflammation causes dysfunctions of the GI tract that, in the context of PD, have been associated with the genesis of constipation and pain.

In the last decade, several works in the field of Neurogastroenterology have demonstrated enteric glia as a modulator cell of enteric neurotransmission, subject to neurotransmitter receptor signaling which generates functional effects on enteric neural reflexes (Seguella and Gulbransen, 2021; Thomasi and Gulbransen, 2023). The myenteric glia, present inside the myenteric plexus, has a privileged location for interaction with enteric neurons (Gabella, 2022). They establish bidirectional communication based on the release of neuroactive substances, neurotransmitters, hemichannels signaling, and neuroimmune mediators which may strongly account for enteric neuroplasticity (Thomasi and Gulbransen, 2023). Enteric glia can be activated by cholinergic signaling, the main excitatory control pathway of the ENS, to release neurotransmitters such as GABA and ATP, in addition to neuromodulators such as prostaglandins, cytokines, and trophic factors (Esposito et al., 2017; Fried et al., 2017; Delvalle et al., 2018b). Intercellular glial signaling via Connexin-43 (Cx43) is described as a mediator of neural reflexes and its absence in animal models knockouts impact colonic migrating motor complexes (McClain et al., 2014; Grubisic and Parpura, 2017). Furthermore, enteric glial activation modulates and activates enteric neural reflexes (McClain et al., 2015; Ahmadzai et al., 2021). The description of glial reactivity in the intestine of patients with PD and the early inflammatory scenario reinforces the idea that impaired glial cell activity may be associated with dysmotility manifested as constipation in PD. Abnormalities in glial signaling, decrease or loss of these cells, or even a pro-inflammatory polarization are considered possible mechanisms of gliopathy associated with constipation (Bassotti et al., 2011; Holland et al., 2021). Considering the progression of PD, GI dysfunction mediated and/or modulated by the reactive profile of enteric glia, probably reinforces peripheral pathological processes, such as dysbiosis and neuroinflammation itself.

The pain component is extremely relevant in any pathology because it impairs the patient’s quality of life and, in PD, also because it increases the frequency of other non-motor symptoms, such as depression and sleep disorders (Roversi et al., 2021). Visceral pain is triggered by the activation of nociceptors in the internal organs. Nociceptors are vast ion-channel receptor class specialists in the detection of harmful stimuli. It can be modulated by autonomic and central neural pathways, by immune mediators, and gains its perception in the CNS (Roversi et al., 2021). Among them, the TRPV channel family is commonly described in multiple GI disorders with a prevalence of the multisensory receptor TRPV1 (Du et al., 2019). This receptor is broadly expressed in the neural structures of the ENS as the mucosal layer, myenteric plexus, and mainly in the submucosal plexus (Akbar et al., 2010). The current notion about TRPV1 in the gut wall describes its expression mainly as from extrinsic innervation terminals and by intrinsic sensory neurons directly connecting ENS and CNS in pain pathways (Matsumoto et al., 2009; Delvalle et al., 2018a). Data from patients with PD describe abdominal pain related to GI dysfunction. In accordance with primary constipation, pain commonly precedes PD motor symptoms over many years, and it is not necessarily related to the severity of motor symptoms (Ha and Jankovic, 2012). Likely, disturbed GI immunity at the beginning of PD pathology is another contributor to visceral hypersensitivity in the prodromal phase of PD since several pain-sensitizing ligands from immune cells can activate nociceptors, as TRPV1 (Roversi et al., 2021). Considering the early intestinal neuroinflammatory component and constipation, it is very likely that PD patients experience both abnormal stimulations in the GI wall that sensitize nociception fibers and altered transduction and perception of pain, at the peripheral or central levels (Wong et al., 2020).

In the giant GI surface with intrinsic and extrinsic innervations closing signaling, pathological mechanisms from ENS are likely to operate underlying GI dysfunction-related pain during PD. Using the 6-OHDA animal model of PD, Pellegrini and collaborators showed increased tachykininergic enteric neurotransmission in the colonic ENS after neurodegeneration induction. In addition, inflammation with enteric glia overexpression of GFAP and increased substance P labeling were both detected in the large intestine (Pellegrini et al., 2016b). Tachykinins, as substance P and neurokinins, and their neurokinin receptors (NKR) are broadly found in the GI tract, and they have been described as neuroglial mediators during intestinal inflammation and pain (Corsetti et al., 2015; Delvalle et al., 2018a). Neurokinin A (NKA) is shown to drive enteric neuroglial responses evoked primarily by neurons that recruit surrounding enteric glia through purinergic and Cx43 pathways – known inflammatory-related mechanisms (Delvalle et al., 2018a; Grubisic et al., 2020). Colonic TRPV1+ varicosities express functional NK2Rs, respond to NKA and, prevent glial reactivity when blocked during inflammation (Delvalle et al., 2018a). As seen in the colitis animal model, NK2R blockade also avoided muscle contractibility dysfunction, demonstrating that TRPV1+ neurons with functional NK signaling are associated with reactive glial and dysmotility during inflammation. In a late report, using transgenic animals expressing genetically encoded calcium indicators in TRPV1+ fibers, Cx43 hemichannels were again implicated during intestinal inflammation as a sensitizer mechanism of colonic nociceptors (Grubisic et al., 2020). In addition, specific enteric glia activation through Designer Receptors Exclusively Activated by Designer Drugs, not only modulates visceromotor responses but also potentiates nociceptors activation in sensory neurons in a pro-inflammatory context (Morales-Soto and Gulbransen, 2020). Furthermore, glial Cx43 has been described as a potent modulator of abdominal hypersensitivity during inflammation by modulating macrophage phenotype in the neuromuscular compartment (Grubisic et al., 2020), a region that undergoes profound neurochemical and inflammatory alterations in animal models of PD (Fornai et al., 2016; Pellegrini et al., 2016b; Thomasi et al., 2022). Evaluations on irritable bowel syndrome gut biopsies also point out that both glial S100B and Cx43 play a functional role in the frequency and intensity of pain reported by patients which may count with modified glial responsiveness to purines, a fundamental neuroglial communication mechanism (Lilli et al., 2018).

Animals submitted to nigrostriatal neurodegeneration using 6-OHDA display increased central expression of TRPV1 associated with thermal and mechanical hyperalgesia (Li et al., 2020). Interestingly, in the CNS of animal models of PD, TRPV1 signaling has been linked with neuronal restoration and oxidative stress amelioration, and these processes are associated with glial activation or glial mechanisms (Nam et al., 2015; Chung et al., 2017). No research has been published investigating enteric TRPV1 signaling in the multiple aspects this receptor could be involved in during PD, mainly intestinal inflammation and abdominal pain. Moreover, despite the extensive report about glial roles in the central neuroplasticity in pain pathways, and the ability of the central glial network to convey signals intestine-derived (Gazerani, 2021; He et al., 2021; Hiraga et al., 2022; Wu et al., 2023), we have few descriptions of enteric glia on the gut-brain axis pain signaling during PD despite their likely contribution. In an elegant report, Lucarini and colleagues demonstrated that enteric glial impairment using glial poisoning before colitis induction was enough to contain visceral hypersensitivity and inflammation as well (Lucarini et al., 2021). When enteric glia were inhibited in this scenario, there was also reduced overexpression of S100B and TRPV1 at different points along the gut-brain axis, from the myenteric plexus, gaining the dorsal root ganglia and reaching the periaqueductal gray area – the latter being a relevant brain area for pain perception (Lucarini et al., 2021). Given the potential of enteric glia to act as an (I) local modulator, sensitizing nociceptors, (II) their ability to signal and recruit glial networks in the gut-brain axis, (III) the description of their pathological state in the ENS of patients with PD, enteric glia may be a novel target with innovative potential for the development of therapies related to pain during PD.

## 5. Could enteric glial cells be a therapeutic target for Parkinson’s disease treatment?

The ENS is a target for the treatment of several GI disorders, such as achalasia, slow transit constipation, and gastroparesis (Fleming et al., 2020). Many of these conditions are related to DGBIs. However, there is no current effective treatment for PD-related GI disorders. Likewise, whether therapeutic strategies addressing the gut could also improve CNS-related PD symptoms is unknown. Due to its central role in GI and gut-brain homeostasis, enteric glial cells have emerged as a potential target for DGBI therapy.

Despite drugs or therapies specifically designed to modulate enteric glial cells do not exist, ongoing studies aim to explore enteric glial mechanisms to improve gut functioning (Gulbransen and Christofi, 2018). Indeed, such mechanisms are affected by some available therapy strategies for functional bowel disorders (Seguella and Gulbransen, 2021). Among the main candidates, intracellular glial Ca^2+^ signaling could be highlighted, which influences gut motility and secretion. Downstream glial intracellular Ca^2+^ responses involve Cx43 hemichannels, which also engage in glial-mediated inflammatory activity by releasing ATP and activating P2X7 and pannexin-1 receptors (Gulbransen and Christofi, 2018). Thus, targeting Cx43 could improve both neuroinflammation and dysmotility. Some other glial mediators involved in immunological signaling that are hypothesized to contribute to gut pathological processes could also be targeted by new therapeutic strategies, such as IL-1R, TLR4, RAGE and peroxisome proliferator-activated receptor alpha (PPAR-α; Esposito et al., 2007, 2014; Stoffels et al., 2014).

Enteric glia are a predominant source of GDNF, a trophic factor that has pivotal protective effects on ENS and IEB (Meir et al., 2021). GDNF levels are altered in disorders characterized by GI neuroinflammation, such as IBDs, Hirschsprung disease and PD (Ochoa-Cortes et al., 2016; Manfredsson et al., 2020) and it has been shown to improve colonic functions and induce enteric neurogenesis in Hirschsprung disease mouse models (Soret et al., 2020). GDNF-based therapy is currently being discussed as an alternative treatment for PD. Although successfully tested in preclinical models, it has failed to reproduce motor symptom amelioration in patients (Marks et al., 2008; Merola et al., 2020). Still, researchers in the field believe the failure is mainly related to inappropriate delivery rather than GDNF ineffectiveness (Manfredsson et al., 2020). Nonetheless, all studies focus on CNS modulation and whether and how it would affect ENS and gut function is unknown. Given the previously described changes in GDNF gut levels, neuroinflammation and increased IEB permeability during PD, it is very likely that a GDNF-based therapy would impact PD GI symptoms by modulating enteric glia function toward an anti-inflammatory profile, among other gut mechanisms (Pellegrini et al., 2020; Thomasi et al., 2022).

More recently, some gut-oriented therapies have been proposed for treating PD symptoms, of which the use of nutraceuticals and the modulation of gut microbiota could be highlighted as the most promising strategies. Nutraceutical is a substance or part of a food that is beneficial for health and can be used for medical purposes, including disease prevention and treatment. Although available data is scarce, some studies suggest these compounds might have antioxidant, anti-inflammatory, or modulatory effects on enteric glial cells. For example, compounds like palmitoylethanolamide, cannabidiol and berberine can reduce gut inflammation and nociception in both IBD models and patients (López-Gómez et al., 2021). For the first two, these effects were described to occur through the activation of PPARs in enteric glial cells (Filippis et al., 2011; Sarnelli et al., 2018). The use of nutraceuticals that modulate glial cells as a therapeutic strategy was also investigated in a neurotoxin-based model of PD with peripheral induction. Antioxidant coffee compounds prevented nigrostriatal and enteric neurodegeneration by enhancing the antioxidant properties of glial cells after rotenone subcutaneous administration (Miyazaki et al., 2019). However, the study did not evaluate whether it could also improve gut symptoms, a known feature of the rotenone PD models. Given that inflammation is one of the pathological events underlying PD GI dysfunction, nutraceutical compounds that can target the enteric glia should be considered in future studies as a potential therapy for PD-related gut dysfunction.

The enteric glia can also be indirectly targeted as a therapeutic strategy by modulating the gut microbiota. The gut microbiota can be modulated in many ways, including nutraceuticals such as probiotics. Other strategies include prebiotics, antibiotics, and fecal microbiota transplantation (FMT), which might affect glial mechanisms in the gut (Xu et al., 2022). For example, the administration of *Bifidobacterium bifidum* in a model of intestinal inflammation could reduce gut inflammation by regulating the release of pro-inflammatory molecules by the enteric glia (Yang et al., 2020). Gut microbiota modulation has been intensively investigated as a therapy for PD, with some successful tests in both human and preclinical studies (Xu et al., 2022; DuPont et al., 2023). In a neurotoxin model of PD, FMT reduced neuroinflammation by decreasing both microglia and astrocyte activation, as well as TLR4/TNF-a signaling pathway (Sun et al., 2018; Zhao et al., 2021). The benefits of the treatment were attributed to the increase in the presence of SCFAs, however, GI pathophysiology was not addressed. Likewise, sodium butyrate was further reported to restore IEB integrity, reduce systemic inflammation and improve motor behavior in the same PD model (Xu et al., 2022). However, whether the mechanisms of proposed microbiota-based PD therapies involve enteric glia is still unknown, yet likely. Despite promising, therapies targeting the gut microbiota still have many challenges to be established as a reliable treatment for PD symptoms, including the great variability of microbiota among patients, variability of diets, which also influences microbiota composition, and a better understanding of the underlying mechanisms. Further studies could address the effects of microbiota modulation on the enteric glia during PD and how it could relate to the improvement of both GI and motor dysfunction.

## 6. Conclusion

**Figure 1:**
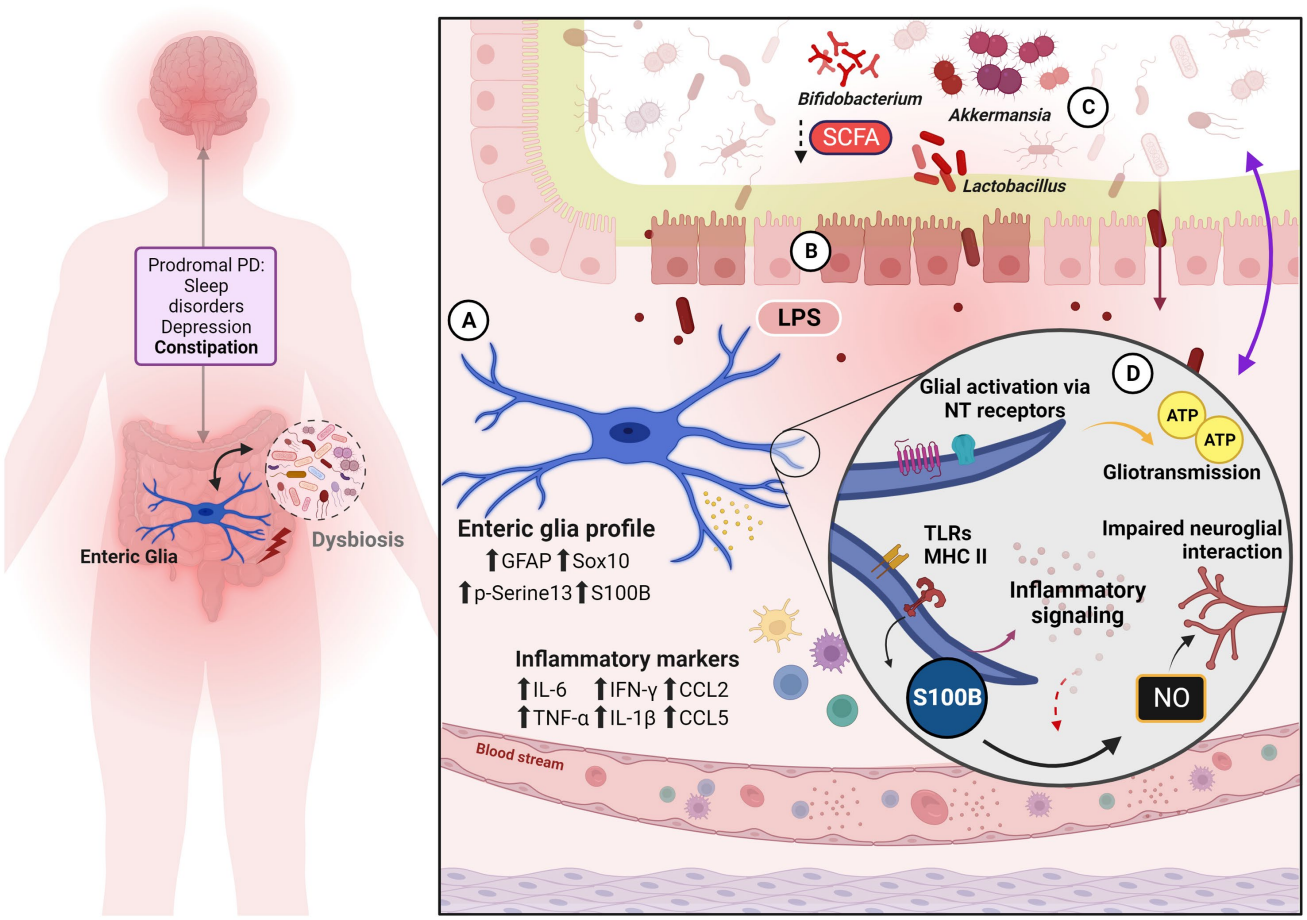
Enteric glia, gut dysfunction and neuroimmune mechanisms to gut-brain axis sensitization. On the left, are the classic pre-motor and non-motor symptoms of the prodromal phase of PD. Both glial reactivity and inflammation are strongly implicated in the neurodegenerative process and contribute to pathology progression. Dysbiosis adds to this scenario as another pro-inflammatory element. **(A)** Enteric glia phenotype and inflammatory signature verified in biopsy samples from PD patients and PD animal models. **(B)** The condition of leaky gut is evidenced by the disruption of the IEB, given the alteration of the tight junction, thus promoting an increase in intestinal permeability—which should reinforce, reciprocally, the intestinal dysbiosis observed during PD. Subsequent immune activation is one of the gut-brain axis signaling pathways. **(C)** Exposure to bacterial bioproducts as well as the reduction of microbe-derived beneficial factors (such as SCFA) to the nervous system, reinforce the pro-inflammatory condition and modify the axis based on signaling by biofilm and its derivatives. **(D)** Enteric glial mechanisms are recognized in PD and/or IBD associated with pro-inflammatory action, dysmotility, and visceral pain.

The gut-brain axis sensitization in PD and enteric glia roles in this scenario are summarized in Figure 1. PD is a complex neurodegenerative disorder involving significant gut-brain axis dysregulation. Interaction between intestinal, neural, and immunological components are key factors in the pathology of PD. PD-related GI symptoms, including constipation and abdominal pain, are emerging as early indicators of the disease, and the involvement of enteric glial cells in the pathogenesis of these symptoms presents them as potential therapeutic targets. Currently, there are no effective treatments that target both PD GI and motor symptoms, despite significant research efforts. Considering the complex bidirectional interactions between the gut and the brain, enteric glia represent a promising target for therapeutic intervention in PD because of their immunomodulatory ability. Glia-oriented therapies could provide anti-inflammatory and antioxidant outcomes and trophic responses as well. By focusing on modulating these cells, therapeutical strategies that address both the GI and central aspects of the disease in an early period may impact the pathologic course, symptoms development, and quality of life of patients with PD.

## Author contributions

BT: Conceptualization, Supervision, Writing – original draft, Writing – review & editing. LV: Writing – original draft, Writing – review & editing. MR: Writing – review & editing. MG: Writing – review & editing. IF: Writing – review & editing. AT-G: Conceptualization, Funding acquisition, Supervision, Writing – original draft, Writing – review & editing.

## References

[ref1] Ahmadzai M. M. Seguella L. Gulbransen B. D. (2021). Circuit-specific enteric glia regulate intestinal motor neurocircuits. Proc. Natl. Acad. Sci. U. S. A. 118:e2025938118. doi: 10.1073/pnas.2025938118, PMID: 34593632 PMC8501758

[ref2] Akbar A. Yiangou Y. Facer P. Brydon W. G. Walters J. R. F. Anand P.. (2010). Expression of the TRPV1 receptor differs in quiescent inflammatory bowel disease with or without abdominal pain. Gut 59, 767–774. doi: 10.1136/gut.2009.194449 20551462

[ref3] Angelopoulou E. Paudel Y. N. Piperi C. (2021). Emerging role of S100B protein implication in Parkinson’s disease pathogenesis. Cell. Mol. Life Sci. 78, 1445–1453. doi: 10.1007/s00018-020-03673-x 33052436 PMC11073186

[ref4] Anselmi L. Toti L. Bove C. Hampton J. Travagli R. A. (2017). A Nigro-vagal pathway controls gastric motility and is affected in a rat model of parkinsonism. Gastroenterology 153, 1581–1593. doi: 10.1053/j.gastro.2017.08.069, PMID: 28912019 PMC5705565

[ref5] Bassotti G. Villanacci V. Nejad M. R. (2011). Chronic constipation: no more idiopathic, but a true neuropathological entity. Gastroenterol Hepatol Bed Bench 4, 109–115. 24834167 PMC4017417

[ref6] Blandini F. Balestra B. Levandis G. Cervio M. Greco R. Tassorelli C.. (2009). Functional and neurochemical changes of the gastrointestinal tract in a rodent model of Parkinson’s disease. Neurosci. Lett. 467, 203–207. doi: 10.1016/j.neulet.2009.10.035 19835930

[ref7] Bohorquez D. V. Samsa L. A. Roholt A. Medicetty S. Chandra R. Liddle R. A. (2014). An enteroendocrine cell-enteric glia connection revealed by 3D electron microscopy. PloS One 9:e89881. doi: 10.1371/journal.pone.0089881, PMID: 24587096 PMC3935946

[ref8] Borghammer P. Berge N. V. D. (2019). Brain-first versus gut-first Parkinson’s disease: a hypothesis. J. Parkinsons Dis. 9, S281–S295. doi: 10.3233/jpd-191721, PMID: 31498132 PMC6839496

[ref9] Borghammer P. Just M. K. Horsager J. Skjærbæk C. Raunio A. Kok E. H.. (2022). A postmortem study suggests a revision of the dual-hit hypothesis of Parkinson’s disease. NPJ Parkinsons Dis. 8:166. doi: 10.1038/s41531-022-00436-2 36450732 PMC9712280

[ref10] Carabotti M. Scirocco A. Maselli M. A. Severi C. (2015). The gut-brain axis: interactions between enteric microbiota, central and enteric nervous systems. Ann. Gastroenterol. 28, 203–209. 25830558 PMC4367209

[ref11] Carrasco A. J. P. Timmermann L. Pedrosa D. J. (2018). Management of constipation in patients with Parkinson’s disease. NPJ Parkinsons Dis. 4:6. doi: 10.1038/s41531-018-0042-8, PMID: 29560414 PMC5856748

[ref12] Cheadle G. A. Costantini T. W. Bansal V. Eliceiri B. P. Coimbra R. (2014). Cholinergic signaling in the gut: a novel mechanism of barrier protection through activation of enteric glia cells. Surg. Infect. (Larchmt.) 15, 387–393. doi: 10.1089/sur.2013.103, PMID: 24828283

[ref13] Chen S.-J. Lin C.-H. (2022). Gut microenvironmental changes as a potential trigger in Parkinson’s disease through the gut–brain axis. J. Biomed. Sci. 29:54. doi: 10.1186/s12929-022-00839-6 35897024 PMC9327249

[ref14] Chow A. K. Gulbransen B. D. (2017). Potential roles of enteric glia in bridging neuroimmune communication in the gut. Am. J. Physiol. Gastrointest. Liver Physiol. 312, G145–G152. doi: 10.1152/ajpgi.00384.2016, PMID: 28039160 PMC5338608

[ref15] Chung Y. C. Baek J. Y. Kim S. R. Ko H. W. Bok E. Shin W.-H.. (2017). Capsaicin prevents degeneration of dopamine neurons by inhibiting glial activation and oxidative stress in the MPTP model of Parkinson’s disease. Exp. Mol. Med. 49:e298. doi: 10.1038/emm.2016.159, PMID: 28255166 PMC5382554

[ref16] Clairembault T. Kamphuis W. Leclair-Visonneau L. Rolli-Derkinderen M. Coron E. Neunlist M.. (2014). Enteric GFAP expression and phosphorylation in Parkinson’s disease. J. Neurochem. 130, 805–815. doi: 10.1111/jnc.12742 24749759

[ref17] Clairembault T. Leclair-Visonneau L. Coron E. Bourreille A. Dily S. L. Vavasseur F.. (2015). Structural alterations of the intestinal epithelial barrier in Parkinson’s disease. Acta Neuropathol. Commun. 3:12. doi: 10.1186/s40478-015-0196-0, PMID: 25775153 PMC4353469

[ref18] Coletto E. Tough I. R. Pritchard S. Hikima A. Jackson M. J. Jenner P.. (2021). Dysregulation of epithelial ion transport and neurochemical changes in the colon of a parkinsonian primate. NPJ Parkinsons Dis 7:9. doi: 10.1038/s41531-020-00150-x, PMID: 33479243 PMC7820491

[ref19] Colucci M. Cervio M. Faniglione M. Angelis S. D. Pajoro M. Levandis G.. (2012). Intestinal dysmotility and enteric neurochemical changes in a Parkinson’s disease rat model. Auton. Neurosci. 169, 77–86. doi: 10.1016/j.autneu.2012.04.005 22608184

[ref20] Corbille A. G. Clairembault T. Coron E. Leclair-Visonneau L. Preterre C. Neunlist M.. (2016). What a gastrointestinal biopsy can tell us about Parkinson’s disease? Neurogastroenterol. Motil. 28, 966–974. doi: 10.1111/nmo.12797, PMID: 26914487

[ref21] Corsetti M. Akyuz F. Tack J. (2015). Targeting tachykinin receptors for the treatment of functional gastrointestinal disorders with a focus on irritable bowel syndrome. Neurogastroenterol. Motil. 27, 1354–1370. doi: 10.1111/nmo.12616 26088804

[ref22] D’Antongiovanni V. Pellegrini C. Antonioli L. Ippolito C. Segnani C. Benvenuti L.. (2023). Enteric glia and brain Astroglia: complex communication in health and disease along the gut-brain Axis. Neuroscience 13:107385842311634. doi: 10.1177/10738584231163460, PMID: 37052336

[ref23] Da Silveira A. B. Oliveira E. C. De Neto S. G. Luquetti A. O. Fujiwara R. T. Oliveira R. C.. (2011). Enteroglial cells act as antigen-presenting cells in chagasic megacolon. Hum. Pathol. 42, 522–532. doi: 10.1016/j.humpath.2010.06.016 21208643

[ref24] Defries D. Beltran M. (2020). Short chain fatty acid transporter/receptor expression and signaling in enteric glial cells. Curr. Dev. Nutr. 4, nzaa057_017–nzaa057_1201. doi: 10.1093/cdn/nzaa057_017

[ref25] Delvalle N. M. Dharshika C. Morales-Soto W. Fried D. E. Gaudette L. Gulbransen B. D. (2018a). Communication between enteric neurons, glia, and nociceptors underlies the effects of tachykinins on Neuroinflammation. Cell. Mol. Gastroenterol. Hepatol. 6, 321–344. doi: 10.1016/j.jcmgh.2018.05.009, PMID: 30116771 PMC6091443

[ref26] Delvalle N. M. Fried D. E. Rivera-Lopez G. Gaudette L. Gulbransen B. D. (2018b). Cholinergic activation of enteric glia is a physiological mechanism that contributes to the regulation of gastrointestinal motility. Am. J. Physiol. Gastrointest. Liver Physiol. 315, G473–G483. doi: 10.1152/ajpgi.00155.2018, PMID: 29927320 PMC6230698

[ref27] Devos D. Lebouvier T. Lardeux B. Biraud M. Rouaud T. Pouclet H.. (2013). Colonic inflammation in Parkinson’s disease. Neurobiol. Dis. 50, 42–48. doi: 10.1016/j.nbd.2012.09.007 23017648

[ref28] Di Vito R. Conte C. Traina G. (2022). A multi-strain probiotic formulation improves intestinal barrier function by the modulation of tight and adherent junction proteins. Cells 11:2617. doi: 10.3390/cells11162617, PMID: 36010692 PMC9406415

[ref29] Dodiya H. B. Forsyth C. B. Voigt R. M. Engen P. A. Patel J. Shaikh M.. (2020). Chronic stress-induced gut dysfunction exacerbates Parkinson’s disease phenotype and pathology in a rotenone-induced mouse model of Parkinson’s disease. Neurobiol. Dis. 135:104352. doi: 10.1016/j.nbd.2018.12.012, PMID: 30579705

[ref30] Du Q. Liao Q. Chen C. Yang X. Xie R. Xu J. (2019). The role of transient receptor potential Vanilloid 1 in common diseases of the digestive tract and the cardiovascular and respiratory system. Front. Physiol. 10:1064. doi: 10.3389/fphys.2019.01064, PMID: 31496955 PMC6712094

[ref31] DuPont H. L. Suescun J. Jiang Z.-D. Brown E. L. Essigmann H. T. Alexander A. S.. (2023). Fecal microbiota transplantation in Parkinson’s disease—a randomized repeat-dose, placebo-controlled clinical pilot study. Front. Neurol. 14:1104759. doi: 10.3389/fneur.2023.1104759, PMID: 36937520 PMC10019775

[ref32] Esposito G. Capoccia E. Gigli S. Pesce M. Bruzzese E. D’Alessandro A.. (2017). HIV-1 tat-induced diarrhea evokes an enteric glia-dependent neuroinflammatory response in the central nervous system. Sci. Rep. 7:7735. doi: 10.1038/s41598-017-05245-9, PMID: 28798420 PMC5552820

[ref33] Esposito G. Capoccia E. Turco F. Palumbo I. Lu J. Steardo A.. (2014). Palmitoylethanolamide improves colon inflammation through an enteric glia/toll like receptor 4-dependent PPAR-alpha activation. Gut 63, 1300–1312. doi: 10.1136/gutjnl-2013-305005, PMID: 24082036

[ref34] Esposito G. Cirillo C. Sarnelli G. Filippis D. D. D’Armiento F. P. Rocco A.. (2007). Enteric glial-derived S100B protein stimulates nitric oxide production in celiac disease. Gastroenterology 133, 918–925. doi: 10.1053/j.gastro.2007.06.009, PMID: 17678654

[ref35] Filippis D. D. Esposito G. Cirillo C. Cipriano M. Winter B. Y. D. Scuderi C.. (2011). Cannabidiol reduces intestinal inflammation through the control of Neuroimmune Axis. PloS One 6:e28159. doi: 10.1371/journal.pone.0028159, PMID: 22163000 PMC3232190

[ref36] Fitzgerald E. Murphy S. Martinson H. A. (2019). Alpha-Synuclein pathology and the role of the microbiota in Parkinson's disease. Front. Neurosci. 13:369. doi: 10.3389/fnins.2019.00369, PMID: 31068777 PMC6491838

[ref37] Flamant M. Aubert P. Rolli-Derkinderen M. Bourreille A. Neunlist M. R. Mahe M. M.. (2011). Enteric glia protect against *Shigella flexneri* invasion in intestinal epithelial cells: a role for S-nitrosoglutathione. Gut 60, 473–484. doi: 10.1136/gut.2010.229237, PMID: 21139062

[ref38] Fleming M. A. Ehsan L. Moore S. R. Levin D. E. (2020). The enteric nervous system and its emerging role as a therapeutic target. Gastroenterol. Res. Pract. 2020, 8024171–8024113. doi: 10.1155/2020/8024171, PMID: 32963521 PMC7495222

[ref39] Fornai M. Pellegrini C. Antonioli L. Segnani C. Ippolito C. Barocelli E.. (2016). Enteric dysfunctions in experimental Parkinson’s disease: alterations of excitatory cholinergic neurotransmission regulating colonic motility in rats. J. Pharmacol. Exp. Ther. 356, 434–444. doi: 10.1124/jpet.115.228510, PMID: 26582732

[ref40] Forsyth C. B. Shannon K. M. Kordower J. H. Voigt R. M. Shaikh M. Jaglin J. A.. (2011). Increased intestinal permeability correlates with sigmoid mucosa alpha-synuclein staining and endotoxin exposure markers in early Parkinson’s disease. PloS One 6:e28032. doi: 10.1371/journal.pone.0028032, PMID: 22145021 PMC3228722

[ref41] Fried D. E. Watson R. E. Robson S. C. Gulbransen B. D. (2017). Ammonia modifies enteric neuromuscular transmission through glial γ-aminobutyric acid signaling. Am. J. Physiol. Gastrointest. Liver Physiol. 313, G570–G580. doi: 10.1152/ajpgi.00154.2017, PMID: 28838986 PMC5814673

[ref42] Gabella G. (2022). Enteric glia: extent, cohesion, axonal contacts, membrane separations and mitochondria in Auerbach’s ganglia of guinea pigs. Cell Tissue Res. 389, 409–426. doi: 10.1007/s00441-022-03656-3, PMID: 35729372 PMC9436829

[ref43] Garrido-Gil P. Rodriguez-Perez A. I. Dominguez-Meijide A. Guerra M. J. Labandeira-Garcia J. L. (2018). Bidirectional neural interaction between central dopaminergic and gut lesions in Parkinson’s disease models. Mol. Neurobiol. 55, 7297–7316. doi: 10.1007/s12035-018-0937-8, PMID: 29404956

[ref44] Gazerani P. (2021). Satellite glial cells in pain research: a targeted viewpoint of potential and future directions. Front. Pain Res. 2:646068. doi: 10.3389/fpain.2021.646068, PMID: 35295432 PMC8915641

[ref45] Gorecki A. M. Anyaegbu C. C. Anderton R. S. (2021). TLR2 and TLR4 in Parkinson’s disease pathogenesis: the environment takes a toll on the gut. Transl. Neurodegener. 10:47. doi: 10.1186/s40035-021-00271-0, PMID: 34814947 PMC8609261

[ref46] Grubišić V. Bali V. Fried D. E. Eltzschig H. K. Robson S. C. Mazei-Robison M. S.. (2022). Enteric glial adenosine 2B receptor signaling mediates persistent epithelial barrier dysfunction following acute DSS colitis. Mucosal Immunol. 15, 964–976. doi: 10.1038/s41385-022-00550-7, PMID: 35869148 PMC9385475

[ref47] Grubisic V. McClain J. L. Fried D. E. Grants I. Rajasekhar P. Csizmadia E.. (2020). Enteric glia modulate macrophage phenotype and visceral sensitivity following inflammation. Cell Rep. 32:108100. doi: 10.1016/j.celrep.2020.108100, PMID: 32905782 PMC7518300

[ref48] Grubisic V. Parpura V. (2017). Two modes of enteric gliotransmission differentially affect gut physiology. Glia 65, 699–711. doi: 10.1002/glia.23121, PMID: 28168732 PMC5357187

[ref49] Gulbransen B. D. Bashashati M. Hirota S. A. Gui X. Roberts J. A. MacDonald J. A.. (2012). Activation of neuronal P2X7 receptor-pannexin-1 mediates death of enteric neurons during colitis. Nat. Med. 18, 600–604. doi: 10.1038/nm.2679, PMID: 22426419 PMC3321107

[ref50] Gulbransen B. D. Christofi F. L. (2018). Are we close to targeting enteric glia in gastrointestinal diseases and motility disorders? Gastroenterology 155, 245–251. doi: 10.1053/j.gastro.2018.06.050, PMID: 29964042 PMC6452442

[ref51] Ha A. D. Jankovic J. (2012). Pain in Parkinson’s disease. Mov. Disord. 27, 485–491. doi: 10.1002/mds.23959 21953990

[ref52] He X. Li L. Xian W. Li M. Zhang L. Xu J.. (2021). Chronic colitis exacerbates NLRP3-dependent neuroinflammation and cognitive impairment in middle-aged brain. J. Neuroinflammation 18:153. doi: 10.1186/s12974-021-02199-8, PMID: 34229722 PMC8262017

[ref53] He J. Zhang P. Shen L. Niu L. Tan Y. Chen L.. (2020). Short-Chain Fatty Acids and Their Association with Signalling Pathways in Inflammation, Glucose and Lipid Metabolism. Int. J. Mol. Sci. 21:356. doi: 10.3390/ijms21176356 32887215 PMC7503625

[ref54] Hiraga S. Itokazu T. Nishibe M. Yamashita T. (2022). Neuroplasticity related to chronic pain and its modulation by microglia. Inflamm. Regen. 42:15. doi: 10.1186/s41232-022-00199-6, PMID: 35501933 PMC9063368

[ref55] Holland A. M. Bon-Frauches A. C. Keszthelyi D. Melotte V. Boesmans W. (2021). The enteric nervous system in gastrointestinal disease etiology. Cell. Mol. Life Sci. 78, 4713–4733. doi: 10.1007/s00018-021-03812-y, PMID: 33770200 PMC8195951

[ref56] Horsager J. Knudsen K. Sommerauer M. (2022). Clinical and imaging evidence of brain-first and body-first Parkinson’s disease. Neurobiol. Dis. 164:105626. doi: 10.1016/j.nbd.2022.105626 35031485

[ref57] Houser M. C. Tansey M. G. (2017). The gut-brain axis: is intestinal inflammation a silent driver of Parkinson’s disease pathogenesis? NPJ Parkinsons Dis 3:3. doi: 10.1038/s41531-016-0002-0, PMID: 28649603 PMC5445611

[ref58] Huang B. Chau S. W. H. Liu Y. Chan J. W. Y. Wang J. Ma S. L.. (2023). Gut microbiome dysbiosis across early Parkinson’s disease, REM sleep behavior disorder and their first-degree relatives. Nat. Commun. 14:2501. doi: 10.1038/s41467-023-38248-4, PMID: 37130861 PMC10154387

[ref59] Inlender T. Nissim-Eliraz E. Stavely R. Hotta R. Goldstein A. M. Yagel S.. (2021). Homeostasis of mucosal glial cells in human gut is independent of microbiota. Sci. Rep. 11:12796. doi: 10.1038/s41598-021-92384-9, PMID: 34140608 PMC8211706

[ref60] Kabouridis P. S. Lasrado R. McCallum S. Chng S. H. Snippert H. J. Clevers H.. (2015). Microbiota controls the homeostasis of glial cells in the gut lamina propria. Neuron 85, 289–295. doi: 10.1016/j.neuron.2014.12.037, PMID: 25578362 PMC4306542

[ref61] Kakoty V. K C S. Dubey S. K. Yang C. H. Kesharwani P. Taliyan R. (2021). The gut-brain connection in the pathogenicity of Parkinson disease: putative role of autophagy. Neurosci. Lett. 753:135865. doi: 10.1016/j.neulet.2021.135865, PMID: 33812929

[ref62] Keshavarzian A. Green S. J. Engen P. A. Voigt R. M. Naqib A. Forsyth C. B.. (2015). Colonic bacterial composition in Parkinson’s disease. Mov. Disord. 30, 1351–1360. doi: 10.1002/mds.26307, PMID: 26179554

[ref63] Kim J. Han J.-Y. Lee Y. Kim K. Choi Y. P. Chae S.. (2023). Genetic deletion of nitric oxide synthase 2 ameliorates Parkinson’s disease pathology and neuroinflammation in a transgenic mouse model of synucleinopathy. Mol. Brain 16:7. doi: 10.1186/s13041-023-00996-1, PMID: 36647152 PMC9841612

[ref64] Kishimoto Y. Zhu W. Hosoda W. Sen J. M. Mattson M. P. (2019). Chronic mild gut inflammation accelerates brain neuropathology and motor dysfunction in α-Synuclein mutant mice. Neuromolecular Med. 21, 239–249. doi: 10.1007/s12017-019-08539-5, PMID: 31079293 PMC6701950

[ref65] Klingelhoefer L. Reichmann H. (2017). Chapter twenty-six the gut and nonmotor symptoms in Parkinson’s disease. Int. Rev. Neurobiol. 134, 787–809. doi: 10.1016/bs.irn.2017.05.027 28805583

[ref66] Langness S. Kojima M. Coimbra R. Eliceiri B. P. Costantini T. W. (2017). Enteric glia cells are critical to limiting the intestinal inflammatory response after injury. Am. J. Physiol. Gastrointest. Liver Physiol. 312, G274–G282. doi: 10.1152/ajpgi.00371.2016, PMID: 28082286

[ref67] Leclair-Visonneau L. Neunlist M. Derkinderen P. Lebouvier T. (2020). The gut in Parkinson’s disease: bottom-up, top-down, or neither? Neurogastroenterol. Motil. 32:e13777. doi: 10.1111/nmo.13777, PMID: 31854093

[ref68] Lee H.-S. Lobbestael E. Vermeire S. Sabino J. Cleynen I. (2021). Inflammatory bowel disease and Parkinson’s disease: common pathophysiological links. Gut 70, gutjnl-2020-322429–gutjnl-2020-322417. doi: 10.1136/gutjnl-2020-322429, PMID: 33067333

[ref69] Li T. Chu C. Yu L. Zhai Q. Wang S. Zhao J.. (2022). Neuroprotective effects of *Bifidobacterium breve* CCFM1067 in MPTP-induced mouse models of Parkinson’s disease. Nutrients 14:4678. doi: 10.3390/nu14214678, PMID: 36364939 PMC9655354

[ref70] Li H. Zhang C. Zhang K. Liu Y. Peng X. Zong Q. (2023). Inflammatory bowel disease and risk of Parkinson’s disease: evidence from a meta-analysis of 14 studies involving more than 13.4 million individuals. Front. Med. 10:1137366. doi: 10.3389/fmed.2023.1137366, PMID: 37153103 PMC10157095

[ref71] Li M. Zhu M. Xu Q. Ding F. Tian Y. Zhang M. (2020). Sensation of TRPV1 via 5-hydroxytryptamine signaling modulates pain hypersensitivity in a 6-hydroxydopamine induced mice model of Parkinson’s disease. Biochem. Biophys. Res. Commun. 521, 868–873. doi: 10.1016/j.bbrc.2019.10.204, PMID: 31708101

[ref72] Lilli N. L. Quénéhervé L. Haddara S. Brochard C. Aubert P. Rolli-Derkinderen M.. (2018). Glioplasticity in irritable bowel syndrome. Neurogastroenterol. Motil. 30:e13232. doi: 10.1111/nmo.13232, PMID: 29027719

[ref73] Lin C. Lin H. Ho E. Ke Y. Cheng M. Shiue C.. (2022). Mild chronic colitis triggers parkinsonism in LRRK2 mutant mice through activating TNF-α pathway. Mov. Disord. 37, 745–757. doi: 10.1002/mds.28890, PMID: 34918781

[ref74] Liu W. Zhou T. Tian J. Yu X. Ren C. Cao Z.. (2022). Role of GDNF, GFRα1 and GFAP in a Bifidobacterium-intervention induced mouse model of intestinal neuronal dysplasia. Front. Pediatr. 9:795678. doi: 10.3389/fped.2021.795678, PMID: 35096711 PMC8796853

[ref75] López-Gómez L. Szymaszkiewicz A. Zielińska M. Abalo R. (2021). Nutraceuticals and enteric glial cells. Molecules 26:3762. doi: 10.3390/molecules26123762, PMID: 34205534 PMC8234579

[ref76] Lucarini E. Seguella L. Vincenzi M. Parisio C. Micheli L. Toti A.. (2021). Role of enteric glia as bridging element between gut inflammation and visceral pain consolidation during acute colitis in rats. Biomedicine 9:1671. doi: 10.3390/biomedicines9111671 PMC8616000 34829900

[ref77] Mahlknecht P. Seppi K. Poewe W. (2015). The concept of prodromal Parkinson’s disease. J. Parkinsons Dis. 5, 681–697. doi: 10.3233/jpd-150685, PMID: 26485429 PMC4927924

[ref78] Manfredsson F. P. Polinski N. K. Subramanian T. Boulis N. Wakeman D. R. Mandel R. J. (2020). The future of GDNF in Parkinson’s disease. Front. Aging Neurosci. 12:593572. doi: 10.3389/fnagi.2020.593572, PMID: 33364933 PMC7750181

[ref79] Marks W. J. Ostrem J. L. Verhagen L. Starr P. A. Larson P. S. Bakay R. A.. (2008). Safety and tolerability of intraputaminal delivery of CERE-120 (adeno-associated virus serotype 2–neurturin) to patients with idiopathic Parkinson’s disease: an open-label, phase I trial. Lancet Neurol. 7, 400–408. doi: 10.1016/s1474-4422(08)70065-6, PMID: 18387850

[ref80] Matsumoto K. Kurosawa E. Terui H. Hosoya T. Tashima K. Murayama T.. (2009). Localization of TRPV1 and contractile effect of capsaicin in mouse large intestine: high abundance and sensitivity in rectum and distal colon. *Am. J. Physiol.-Gastrointest*. Liver Physiol. 297, G348–G360. doi: 10.1152/ajpgi.90578.2008 19497956

[ref81] McClain J. L. Fried D. E. Gulbransen B. D. (2015). Agonist-evoked ca(2+) signaling in enteric glia drives neural programs that regulate intestinal motility in mice. Cell. Mol. Gastroenterol. Hepatol. 1, 631–645. doi: 10.1016/j.jcmgh.2015.08.004, PMID: 26693173 PMC4673674

[ref82] McClain J. L. Grubisic V. Fried D. Gomez-Suarez R. A. Leinninger G. M. Sevigny J.. (2014). Ca2+ responses in enteric glia are mediated by connexin-43 hemichannels and modulate colonic transit in mice. Gastroenterology 146:e1, 497–507.e1. doi: 10.1053/j.gastro.2013.10.061, PMID: 24211490 PMC3935238

[ref83] Meir M. Kannapin F. Diefenbacher M. Ghoreishi Y. Kollmann C. Flemming S.. (2021). Intestinal epithelial barrier maturation by enteric glial cells is GDNF-dependent. Int. J. Mol. Sci. 22:1887. doi: 10.3390/ijms22041887, PMID: 33672854 PMC7917776

[ref84] Merola A. Laar A. V. Lonser R. Bankiewicz K. (2020). Gene therapy for Parkinson’s disease: contemporary practice and emerging concepts. Expert Rev. Neurother. 20, 577–590. doi: 10.1080/14737175.2020.1763794, PMID: 32425079

[ref85] Metta V. Leta V. Mrudula K. R. Prashanth L. K. Goyal V. Borgohain R.. (2021). Gastrointestinal dysfunction in Parkinson’s disease: molecular pathology and implications of gut microbiome, probiotics, and fecal microbiota transplantation. J. Neurol. 269, 1154–1163. doi: 10.1007/s00415-021-10567-w, PMID: 33881598

[ref86] Metzdorf J. Tönges L. (2021). Short-chain fatty acids in the context of Parkinson’s disease. Neural Regen. Res. 16, 2015–2016. doi: 10.4103/1673-5374.308089 33642384 PMC8343296

[ref87] Miyazaki I. Isooka N. Wada K. Kikuoka R. Kitamura Y. Asanuma M. (2019). Effects of enteric environmental modification by coffee components on neurodegeneration in rotenone-treated mice. Cells 8:221. doi: 10.3390/cells8030221, PMID: 30866481 PMC6468520

[ref860] Morales-Soto W. Gulbransen B. D. (2020). Enteric Glia Contribute to Visceral Hypersensitivity Through Interactions with Nociceptors. FASEB J. 34:1. doi: 10.1096/fasebj.2020.34.s1.03624

[ref88] Morais L. H. Hara D. B. Bicca M. A. Poli A. Takahashi R. N. (2018). Early signs of colonic inflammation, intestinal dysfunction, and olfactory impairments in the rotenone-induced mouse model of Parkinson’s disease. Behav. Pharmacol. 29, 199–210. doi: 10.1097/fbp.0000000000000389 29543651

[ref89] Moudgal R. Schultz A. W. Shah E. D. (2021). Systemic disease associations with disorders of gut–brain interaction and gastrointestinal transit: a review. Clin. Exp. Gastroenterol. 14, 249–257. doi: 10.2147/ceg.s283685 34135613 PMC8197439

[ref90] Nam J. H. Park E. S. Won S.-Y. Lee Y. A. Kim K. I. Jeong J. Y.. (2015). TRPV1 on astrocytes rescues nigral dopamine neurons in Parkinson’s disease via CNTF. Brain 138, 3610–3622. doi: 10.1093/brain/awv297, PMID: 26490328 PMC4840550

[ref91] Nanni G. Travagli R. A. (2020). A nigro-vagal pathway controls colonic motility and may be impaired in a model of environmental Parkinson’s disease. FASEB J. 34:1. doi: 10.1096/fasebj.2020.34.s1.03531

[ref92] Ochoa-Cortes F. Turco F. Linan-Rico A. Soghomonyan S. Whitaker E. Wehner S.. (2016). Enteric glial cells: a new frontier in Neurogastroenterology and clinical target for inflammatory bowel diseases. Inflamm. Bowel Dis. 22, 433–449. doi: 10.1097/mib.0000000000000667, PMID: 26689598 PMC4718179

[ref93] Orsini M. Liddo R. D. Valeriani F. Mancin M. D’Incà R. Castagnetti A.. (2020). In silico evaluation of putative S100B interacting proteins in healthy and IBD gut microbiota. Cells 9:1697. doi: 10.3390/cells9071697, PMID: 32679810 PMC7407188

[ref94] Palanisamy B. N. Sarkar S. Malovic E. Samidurai M. Charli A. Zenitsky G.. (2022). Environmental neurotoxic pesticide exposure induces gut inflammation and enteric neuronal degeneration by impairing enteric glial mitochondrial function in pesticide models of Parkinson’s disease: potential relevance to gut-brain axis inflammation in Parkinson’s disease pathogenesis. Int. J. Biochem. Cell Biol. 147:106225. doi: 10.1016/j.biocel.2022.106225, PMID: 35550926 PMC10411482

[ref95] Pellegrini C. Colucci R. Antonioli L. Barocelli E. Ballabeni V. Bernardini N.. (2016a). Intestinal dysfunction in Parkinson’s disease: lessons learned from translational studies and experimental models. Neurogastroenterol. Motil. 28, 1781–1791. doi: 10.1111/nmo.12933, PMID: 27611012

[ref96] Pellegrini C. D’Antongiovanni V. Miraglia F. Rota L. Benvenuti L. Salvo C. D.. (2022). Enteric alpha-synuclein impairs intestinal epithelial barrier through caspase-1-inflammasome signaling in Parkinson’s disease before brain pathology. NPJ Parkinsons Dis 8:9. doi: 10.1038/s41531-021-00263-x, PMID: 35022395 PMC8755783

[ref97] Pellegrini C. Fornai M. Colucci R. Tirotta E. Blandini F. Levandis G.. (2016b). Alteration of colonic excitatory tachykininergic motility and enteric inflammation following dopaminergic nigrostriatal neurodegeneration. J. Neuroinflammation 13:146. doi: 10.1186/s12974-016-0608-5, PMID: 27295950 PMC4907252

[ref98] Pellegrini C. Ippolito C. Segnani C. Dolfi A. Errede M. Virgintino D.. (2020). Pathological remodelling of colonic wall following dopaminergic nigrostriatal neurodegeneration. Neurobiol. Dis. 139:104821. doi: 10.1016/j.nbd.2020.104821, PMID: 32088380

[ref99] Perez-Pardo P. Dodiya H. B. Broersen L. M. Douna H. Wijk N. Silva S. L.. (2018). Gut-brain and brain-gut axis in Parkinson’s disease models: Effects of a uridine and fish oil diet. Nutr. Neurosci. 21, 391–402. doi: 10.1080/1028415x.2017.1294555 28276272

[ref100] Perez-Pardo P. Dodiya H. B. Engen P. A. Forsyth C. B. Huschens A. M. Shaikh M.. (2019). Role of TLR4 in the gut-brain axis in Parkinson’s disease: a translational study from men to mice. Gut 68, 829–843. doi: 10.1136/gutjnl-2018-316844, PMID: 30554160

[ref101] Prochera A. Rao M. (2023). Mini-review: enteric glial regulation of the gastrointestinal epithelium. Neurosci. Lett. 805:137215. doi: 10.1016/j.neulet.2023.137215, PMID: 37001854 PMC10125724

[ref102] Rolli-Derkinderen M. Leclair-Visonneau L. Bourreille A. Coron E. Neunlist M. Derkinderen P. (2020). Is Parkinson’s disease a chronic low-grade inflammatory bowel disease? J. Neurol. 267, 2207–2213. doi: 10.1007/s00415-019-09321-0, PMID: 30989372

[ref103] Romano S. Savva G. M. Bedarf J. R. Charles I. G. Hildebrand F. Narbad A. (2021). Meta-analysis of the Parkinson’s disease gut microbiome suggests alterations linked to intestinal inflammation. NPJ Parkinsons Dis 7:27. doi: 10.1038/s41531-021-00156-z, PMID: 33692356 PMC7946946

[ref104] Rosenberg H. J. Rao M. (2021). Enteric glia in homeostasis and disease: from fundamental biology to human pathology. iScience 24:102863. doi: 10.1016/j.isci.2021.102863, PMID: 34401661 PMC8348155

[ref105] Roversi K. Callai-Silva N. Roversi K. Griffith M. Boutopoulos C. Prediger R. D.. (2021). Neuro-immunity and gut Dysbiosis drive Parkinson’s disease-induced pain. Front. Immunol. 12:759679. doi: 10.3389/fimmu.2021.759679, PMID: 34868000 PMC8637106

[ref106] Sampath C. Kalpana R. Ansah T. Charlton C. Hale A. Channon K. M.. (2019). Impairment of Nrf2- and Nitrergic-mediated gastrointestinal motility in an MPTP mouse model of Parkinson’s disease. Dig. Dis. Sci. 64, 3502–3517. doi: 10.1007/s10620-019-05693-5 31187328 PMC6858486

[ref107] Sarnelli G. Seguella L. Pesce M. Lu J. Gigli S. Bruzzese E.. (2018). HIV-1 tat-induced diarrhea is improved by the PPARalpha agonist, palmitoylethanolamide, by suppressing the activation of enteric glia. J. Neuroinflammation 15:94. doi: 10.1186/s12974-018-1126-4, PMID: 29573741 PMC5866515

[ref108] Sarosi G. A. Barnhart D. C. Turner D. J. Mulholland M. W. (1998). Capacitative Ca2+ entry in enteric glia induced by thapsigargin and extracellular ATP. Am. J. Physiol. 275, G550–G555. doi: 10.1152/ajpgi.1998.275.3.g550 9724268

[ref109] Savidge T. C. Newman P. Pothoulakis C. Ruhl A. Neunlist M. Bourreille A.. (2007). Enteric glia regulate intestinal barrier function and inflammation *via* release of S-nitrosoglutathione. Gastroenterology 132, 344–358. doi: 10.1053/j.gastro.2007.01.051 17408650

[ref110] Schneider K. M. Kim J. Bahnsen K. Heuckeroth R. O. Thaiss C. A. (2022). Environmental perception and control of gastrointestinal immunity by the enteric nervous system. Trends Mol. Med. 28, 989–1005. doi: 10.1016/j.molmed.2022.09.005, PMID: 36208986 PMC13734752

[ref111] Seguella L. Capuano R. Sarnelli G. Esposito G. (2019). Play in advance against neurodegeneration: exploring enteric glial cells in gut-brain axis during neurodegenerative diseases. Expert. Rev. Clin. Pharmacol. 12, 555–564. doi: 10.1080/17512433.2019.1612744, PMID: 31025582

[ref112] Seguella L. Gulbransen B. D. (2021). Enteric glial biology, intercellular signalling and roles in gastrointestinal disease. Nat. Rev. Gastroenterol. Hepatol. 18, 571–587. doi: 10.1038/s41575-021-00423-7, PMID: 33731961 PMC8324524

[ref113] Seguella L. Palenca I. Franzin S. B. Zilli A. Esposito G. (2023). Mini-review: interaction between intestinal microbes and enteric glia in health and disease. Neurosci. Lett. 806:137221. doi: 10.1016/j.neulet.2023.137221 37031943

[ref114] Skjærbæk C. Knudsen K. Horsager J. Borghammer P. (2021). Gastrointestinal dysfunction in Parkinson’s disease. J. Clin. Med. 10:493. doi: 10.3390/jcm10030493, PMID: 33572547 PMC7866791

[ref115] Soret R. Schneider S. Bernas G. Christophers B. Souchkova O. Charrier B.. (2020). Glial cell-derived neurotrophic factor induces enteric neurogenesis and improves Colon structure and function in mouse models of Hirschsprung disease. Gastroenterology 159, 1824–1838.e17. doi: 10.1053/j.gastro.2020.07.018, PMID: 32687927

[ref116] Spica V. R. Valeriani F. Orsini M. Clementi M. E. Seguella L. Gianfranceschi G.. (2023). S100B affects gut microbiota biodiversity. Int. J. Mol. Sci. 24:2248. doi: 10.3390/ijms24032248, PMID: 36768570 PMC9916897

[ref117] Stoffels B. Hupa K. J. Snoek S. A. Bree S. Stein K. Schwandt T.. (2014). Postoperative ileus involves interleukin-1 receptor signaling in enteric glia. Gastroenterology 146:e1, 176–87.e1. doi: 10.1053/j.gastro.2013.09.030, PMID: 24067878

[ref118] Sun M.-F. Zhu Y.-L. Zhou Z.-L. Jia X.-B. Xu Y.-D. Yang Q.. (2018). Neuroprotective effects of fecal microbiota transplantation on MPTP-induced Parkinson’s disease mice: gut microbiota, glial reaction and TLR4/TNF-α signaling pathway. Brain Behav. Immun. 70, 48–60. doi: 10.1016/j.bbi.2018.02.005, PMID: 29471030

[ref119] Tan A. H. Hor J. W. Chong C. W. Lim S. (2021). Probiotics for Parkinson’s disease: current evidence and future directions. JGH Open 5, 414–419. doi: 10.1002/jgh3.12450, PMID: 33860090 PMC8035463

[ref120] Tan A. H. Lim S. Y. Lang A. E. (2022). The microbiome–gut–brain axis in Parkinson disease — from basic research to the clinic. Nat. Rev. Neurol. 18, 476–495. doi: 10.1038/s41582-022-00681-2 35750883

[ref121] Tansey M. G. Wallings R. L. Houser M. C. Herrick M. K. Keating C. E. Joers V. (2022). Inflammation and immune dysfunction in Parkinson disease. Nat. Rev. Immunol. 22, 657–673. doi: 10.1038/s41577-022-00684-6, PMID: 35246670 PMC8895080

[ref122] Thomasi B. Gulbransen B. (2023). Mini-review: intercellular communication between enteric glia and neurons. Neurosci. Lett. 806:137263. doi: 10.1016/j.neulet.2023.137263, PMID: 37085112 PMC10150911

[ref123] Thomasi B. B. M. Valdetaro L. Ricciardi M. C. G. Hayashide L. Fernandes A. Mussauer A.. (2022). Enteric glial cell reactivity in colonic layers and mucosal modulation in a mouse model of Parkinson’s disease induced by 6-hydroxydopamine. Brain Res. Bull. 187, 111–121. doi: 10.1016/j.brainresbull.2022.06.013, PMID: 35772606

[ref124] Travagli R. A. Browning K. N. Camilleri M. (2020). Parkinson disease and the gut: new insights into pathogenesis and clinical relevance. Nat. Rev. Gastroenterol. Hepatol. 17, 673–685. doi: 10.1038/s41575-020-0339-z, PMID: 32737460

[ref125] Turco F. Sarnelli G. Cirillo C. Palumbo I. Giorgi F. D. D’Alessandro A.. (2014). Enteroglial-derived S100B protein integrates bacteria-induced toll-like receptor signalling in human enteric glial cells. Gut 63, 105–115. doi: 10.1136/gutjnl-2012-302090, PMID: 23292665

[ref126] Wakabayashi K. Takahashi H. Takeda S. Ohama E. Ikuta F. (1988). Parkinson’s disease: the presence of Lewy bodies in Auerbach’s and Meissner’s plexuses. Acta Neuropathol. 76, 217–221. doi: 10.1007/BF00687767, PMID: 2850698

[ref127] Wong M. Y. W. Hebbard G. Gibson P. R. Burgell R. E. (2020). Chronic constipation and abdominal pain: independent or closely interrelated symptoms? J. Gastroenterol. Hepatol. 35, 1294–1301. doi: 10.1111/jgh.14970 31900961

[ref128] Wu K. Liu Y. Shao S. Song W. Chen X. Dong Y.. (2023). The microglial innate immune receptors TREM-1 and TREM-2 in the anterior cingulate cortex (ACC) drive visceral hypersensitivity and depressive-like behaviors following DSS-induced colitis. Brain Behav. Immun. 112, 96–117. doi: 10.1016/j.bbi.2023.06.003, PMID: 37286175

[ref129] Xing T. Nanni G. Burkholde C. Browning K. Travagli R. A. (2023). The nigro-vagal modulation of the proximal colon is compromised in a model of environmental Parkinson’s Disease. Physiology 38:5733401. doi: 10.1152/physiol.2023.38.s1.5733401

[ref130] Xu R.-C. Miao W.-T. Xu J.-Y. Xu W.-X. Liu M.-R. Ding S.-T.. (2022). Neuroprotective effects of sodium butyrate and monomethyl fumarate treatment through GPR109A modulation and intestinal barrier restoration on PD mice. Nutrients 14:4163. doi: 10.3390/nu14194163 36235813 PMC9571500

[ref131] Yang H. Li S. Le W. (2022). Intestinal permeability, Dysbiosis, inflammation and enteric glia cells: the intestinal etiology of Parkinson’s disease. Aging Dis. 13, 1381–1390. doi: 10.14336/ad.2022.01281, PMID: 36186124 PMC9466983

[ref132] Yang P. Li X. Yang Y. Qian W. Li S. Yan C.. (2020). The influence of Bifidobacterium bifidum and *Bacteroides fragilis* on enteric glial cell–derived neurotrophic factors and Inflammasome. Inflammation 43, 2166–2177. doi: 10.1007/s10753-020-01284-z 32638263

[ref133] Yang Y. Qian W. Hou X. Dai C. (2022). Bifidobacterium bifidum and *Bacteroides fragilis* induced differential immune regulation of enteric glial cells subjected to exogenous inflammatory stimulation. Inflammation 45, 2388–2405. doi: 10.1007/s10753-022-01700-6 35776290

[ref134] Zhang Z. Liu Z. Lv A. Fan C. (2023). How toll-like receptors influence Parkinson’s disease in the microbiome–gut–brain axis. Front. Immunol. 14:1154626. doi: 10.3389/fimmu.2023.1154626, PMID: 37207228 PMC10189046

[ref135] Zhao Z. Ning J. Bao X. Shang M. Ma J. Li G.. (2021). Fecal microbiota transplantation protects rotenone-induced Parkinson’s disease mice via suppressing inflammation mediated by the lipopolysaccharide-TLR4 signaling pathway through the microbiota-gut-brain axis. Microbiome 9:226. doi: 10.1186/s40168-021-01107-9, PMID: 34784980 PMC8597301

